# 7-Ketocholesterol Induces Oxiapoptophagy and Inhibits Osteogenic Differentiation in MC3T3-E1 Cells

**DOI:** 10.3390/cells11182882

**Published:** 2022-09-15

**Authors:** Jing Ouyang, Yaosheng Xiao, Qun Ren, Jishang Huang, Qingluo Zhou, Shanshan Zhang, Linfu Li, Weimei Shi, Zhixi Chen, Longhuo Wu

**Affiliations:** 1 College of Rehabilitation, Gannan Medical University, Ganzhou 341000, China; 2 Department of Orthopedics, First Affiliated Hospital of Gannan Medical University, Ganzhou 341000, China; 3 College of Pharmacy, Gannan Medical University, Ganzhou 341000, China

**Keywords:** oxiapoptophagy, 7-Ketocholesterol, oxidative stress, osteogenic differentiation, osteoporosis

## Abstract

7-Ketocholesterol (7KC) is one of the oxysterols produced by the auto-oxidation of cholesterol during the dysregulation of cholesterol metabolism which has been implicated in the pathological development of osteoporosis (OP). Oxiapoptophagy involving oxidative stress, autophagy, and apoptosis can be induced by 7KC. However, whether 7KC produces negative effects on MC3T3-E1 cells by stimulating oxiapoptophagy is still unclear. In the current study, 7KC was found to significantly decrease the cell viability of MC3T3-E1 cells in a concentration-dependent manner. In addition, 7KC decreased ALP staining and mineralization and down-regulated the protein expression of OPN and RUNX2, inhibiting osteogenic differentiation. 7KC significantly stimulated oxidation and induced autophagy and apoptosis in the cultured MC3T3-E1 cells. Pretreatment with the anti-oxidant acetylcysteine (NAC) could effectively decrease NOX4 and MDA production, enhance SOD activity, ameliorate the expression of autophagy-related factors, decrease apoptotic protein expression, and increase ALP, OPN, and RUNX2 expression, compromising 7KC-induced oxiapoptophagy and osteogenic differentiation inhibition in MC3T3-E1 cells. In summary, 7KC may induce oxiapoptophagy and inhibit osteogenic differentiation in the pathological development of OP.

## 1. Introduction

Osteoporosis (OP), commonly marked by a constant decrease in bone mass and changes in bone/skeletal tissue structure, is a common bone/skeletal disease [1]. More often than not, patients do not realize OP at the early stage until accidental fractural damages occur [2]. The pathogenesis and progression of OP might be affected by aging, genetic factors, bad living habits, and nutritional deficiency, among which, the imbalance of bone homeostasis is one of the key factors [3,4]. There is increasing evidence that lipid metabolism disorders, such as atherosclerosis (AS) and OP, share common underlying pathogenesis involving bone and vascular mineralization, as well as age-related degenerative processes [5,6,7]. However, the specific mechanism of action between lipid and bone metabolism disorders remains unclear.

Oxysterols are the lipid oxidation products of 27C cholesterol obtained from certain diets and cholesterol metabolism by enzymatic or non-enzymatic mechanisms [8]. Endogenous oxysterols are generated by the latter mechanism, with oxidation occurring in the sterol ring. Many factors, such as singlet oxygen and hydrogen peroxide, can induce the oxidation of cholesterol in lipoproteins, cell membranes, and food [9]. Oxysterols can be formed in foods containing cholesterol during prolonged storage or heating. In addition, cholesterol in the stomach can also be transformed into oxidized cholesterol due to an acidic pH and the presence of oxygen and iron ions [10]. Oxysterols can trigger oxidation, inducing pathological damage in some organelles (mitochondria, lysosomes, and peroxisomes) and cell death. Oxysterols have been implicated in important physiological and pathological activities, including cholesterol metabolism homeostasis, cell differentiation and proliferation, inflammation, and OP [11]. Evidence shows that oxidized sterols contribute to atherosclerotic vascular modeling and remodeling, specifically mediating many key steps: dysfunction of endothelial cells, adhesion of circulating blood cells, formation of foam cells and fibrous cap, re-constructure of blood vessels and extracellular matrix (ECM), and cellular apoptosis and unstable plaques [12,13,14,15,16].

7-Ketocholesterol (7KC), a widely studied oxysterol mainly formed by auto-oxidation [17], is often found in industrial foods [18]. 7KC is found to accumulate in the vascular wall, retina, and brain [17]. For example, 7KC is abundant in the circulation of hypercholesterolemia and atherosclerotic lesions [19,20]. 7KC induces oxidative stress, triggers inflammation, and can decrease cell mobility, causing pathological cell injury via aberrant signaling pathways and leading cells to a special form of death. These pathological changes can be referred to as oxiapoptophagy (OXIdative stress + APOPTOsis + autoPHAGY). This form of death is related to the challenges of oxidative stress, apoptosis, and autophagy [21,22,23]. According to previous research, 7KC, 7β-hydroxycholesterol (7β-OHC), and 24(S)-hydroxycholesterol (24S-OHC) have a biological effect on activating oxiapoptophagy [24]. 7KC may exhibit a regulatory activity in macrophage reprogramming and oxiapoptophagy in several cell types/lines [25,26,27,28]. In atherosclerotic plaques, 7KC induces calcium-containing apoptosis and promotes the deposition of cell bodies, which in turn leads to vascular calcification.

Interestingly, 7KC is reported to be an inhibitor of the adipogenic differentiation of adipose-derived stem cells by mediating the Wnt/β-catenin and MAPK signaling pathways [29]. Both adipocytes and osteoblasts can be differentiated from bone marrow-derived mesenchymal stem cells (BMSCs). OP can cause an adipo-osteogenic imbalance and the effects of 7KC on osteogenic differentiation are still unknown. However, it was recently reported that 7KC can promote osteoclast differentiation by increasing the expression of the miR-107-5p/MKP1 axis [30]. Whether 7KC can induce oxiapoptophagy in osteoblasts is still needed for further investigation. In this study, the focus is on the biological actions of 7KC on the induction of oxiapoptophagy and osteogenic differentiation in the cultured pre-osteoblast MC3T3-E1 cells.

## 2. Materials and Methods

### 2.1. Cell Culture

MC3T3-E1 cells were purchased and cultured as in a previous study [7]. Briefly, cells were cultured in α-MEM under standard conditions. An osteogenic induction medium (OIM) was supplemented for 15 days. Cells were pre-incubated with the anti-oxidant N-acetylcysteine (NAC, 2.5 mM, Sigma-Aldrich, St. Louis, MO, USA) overnight and then 7KC (Sigma-Aldrich, St. Louis, MO, USA) with different concentrations (0, 10, 20, and 40 μM) was added. 

### 2.2. Determination of Cell Viability

Cell viability was measured by a CCK-8 kit (Solarbio, Beijing, China). A total of 1 × 10^4^ cells/wells were inoculated on 96-well plates, and then 7KC was added in different concentrations (0, 2.5, 5, 10, 20, and 40 μM). After 24 h or 48 h, the medium was sucked out and cleaned two times with phosphate buffered solution (PBS) (Beyotime), and 10% CCK-8 medium was added to each well. The cells were incubated at 37 °C for 1 h, and then the absorbance was measured at a 450 nm wavelength under the guidelines of a microplate reader (Varioskanlux, Thermo Fisher Scientific, Waltham, MA, USA).

### 2.3. Analysis of ROS Levels

The cells were inoculated with 1 × 10^5^ cells/well in 24-well plates. Then, 500 μL of medium containing different drug concentrations was added to the plates, incubated for 24 h, and washed twice with PBS. The solution contained 10 μM of 2′,7′-Dichlorodihydrofluorescein diacetate (DCFH-DA, Solarbio, Beijing, China) in a serum-free medium and was incubated for 30 min in the dark. The cells were washed in PBS three times. Next, 4% paraformaldehyde (Beyotime, Shanghai, China) was added to the system and the cells were fixed at room temperature for 10 min. After the cells had been washed in PBS three times, 4’,6-diamidino-2-phenylindole (DAPI, Solarbio, Beijing, China) was used for staining for 10 min, the cells were washed three times with PBS again, and the fluorescence intensity was observed using a fluorescence microscope and photographed. ImageJ/Fiji, an open-source image processing package, was used to analyze the mean fluorescence intensity.

### 2.4. Protein Expression Detected by Western Blot

MC3T3-E1 cells (5 × 10^5^ cells/wells) were cultured for 24 h before the experiment. Then, the cells were harvested and lysed on ice in RIPA Lysis Buffer (Beyotime) for 30 min. The lysates were collected after centrifugation and the protein concentration was determined. An electrophoretic separation on 25 µg of each sample was performed by a 10–12% SDS-PAGE gel they were then transferred onto PVDF membranes. After the membranes were incubated for 1 h in TBS plus 5% skim milk, the primary antibodies (all were diluted by 1:1000) anti-NOX4 (Affinity, Jiangsu, China), anti-runx2 (Affinity), anti-LC3-I/II (Affinity), anti-P62 (Affinity), anti-Bax (Affinity), anti-Bcl-2 (Affinity), anti-OPN (Beyotime), anti-Beclin 1 (Beyotime), anti-β-actin (Solarbio), and anti-Cleaved caspase-3 (Cell Signaling Technology, Danvers, MA, USA) were incubated with the membranes at 4 °C overnight. HRP-labeled goat and rabbit secondary antibody (1:5000; Boster, Wuhan, China) was added and incubated at room temperature for 1 h. Finally, protein bands were measured and analyzed by enhanced chemiluminescence detection systems and Fiji ImageJ. 

### 2.5. Apoptosis Analysis

The apoptosis rates were determined by employing the Annexin V apoptosis kit which was obtained from BD Biosciences (San Jose, CA, USA). The cells (5 × 10^5^ cells/wells) were grown until confluence of about 80% was reached. After the proper treatment, the cells were collected. Under the guideline of the kit’s instructions, the cells were moved for apoptotic determination by flow cytometry (BD FACS Canto II, San Jose, CA, USA). 

### 2.6. ALP Staining

MC3T3-E1 cells were inoculated with 1 × 10^5^ cells/wells and cultured for 24 h before experiments. After 14 days of incubation, cells were collected and washed with the Beyotime. The ALP activity was then measured using an ALP activity assay kit (Beyotime). Finally, the absorption at the 405 nm wavelength was measured under the guidelines of a microplate reader (Varioskanlux, Thermo Fisher Scientific, Waltham, MA, USA). 

### 2.7. Determination of MDA and SOD Activities

MC3T3-E1 cells were inoculated with 2 × 10^6^ cells/wells for 24 h before experiments. Then, the cells were harvested and lysed on ice in RIPA Lysis Buffer (Beyotime) for 30 min. The lysates were collected after centrifugation, and the protein concentration was determined. Then, the SOD and MDA activities were measured using a SOD kit (Beijing Solarbio, China) and an MDA kit (Beijing Solarbio, China). Finally, the absorption of SOD and MDA at 560 nm and 532 nm, respectively, was measured under the guidelines of a microplate meter (Varioskanlux). 

### 2.8. Mineralization Detection

MC3T3-E1 cells (1 × 10^5^ cells/well) were cultured for 24 h. After 14 days of incubation, the cells were washed and fixed with 4% paraformaldehyde (Beyotime, Shanghai, China). Then, the cells were stained with alizarin red S (Solarbio, Beijing, China) at 37 °C for 30 min. After washing with PBS, orange calcified nodules were observed under an inverted microscope. Data were analyzed under the guideline of the kit’s instructions. 

### 2.9. Statistical Analysis

Data were indicated as the mean ± standard deviation (SD). Statistical analysis was conducted by the GraphPad Prism v7 software (GraphPad Software Inc., La Jolla, CA, USA). The one-way analysis of variance (ANOVA) and subsequent Bonferroni’s multiple comparisons test were analyzed. *p* < 0.05 indicated a statistical difference.

## 3. Results

### 3.1. 7KC Inhibited the Viability of MC3T3-E1 Cells 

**Figure 1:**
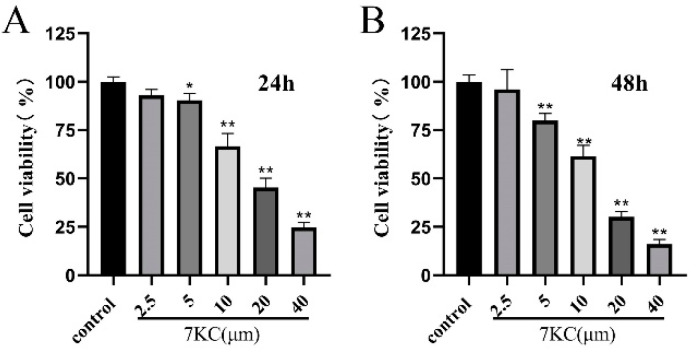
7KC inhibited MC3T3-E1 cell viability. (**A**,**B**) MC3T3-E1 cells were treated with 7KC (0, 2.5, 5, 10, 20, or 40 μM) for 24 h and 48 h. * *p* < 0.05; ** *p* < 0.01.

MC3T3-E1 cell viability at 24 h and 48 h, respectively, after the intervention of 7KC (0, 2.5, 5, 10, 20, or 40 μM), is shown in Figure 1A,B. At concentrations above 5 μM, 7KC significantly decreased MC3T3-E1 cell viability within 24 h or 48 h, compared with that in the control group.

### 3.2. 7KC Inhibited the Osteogenic Differentiation of MC3T3-E1 Cells

**Figure 2:**
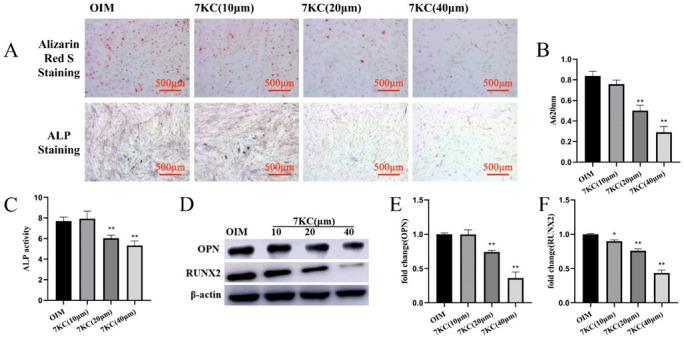
7KC inhibited the osteogenic differentiation of MC3T3-E1 cells. MC3T3-E1 cells were treated with 7KC (0, 10, 20, or 40 μM). (**A**–**C**) ALP activity detection and the mineral assays were performed (×50 magnification); ALP activity detection and quantitative analysis of the mineralized area. The protein expressions of OPN (**D**,**E**) and RUNX2 (**D**,**F**) were analyzed by Western blot. Data were analyzed and compared with the OIM group. * *p* < 0.05; ** *p* < 0.01. OIM, osteogenic induction medium.

In order to explore the biological actions of 7KC on MC3T3-E1 cell differentiation, ALP activity detection and mineral assays were conducted. Results showed that 7KC significantly attenuated ALP activity and alizarin red S staining (Figure 2A–C), suggesting that cell differentiation was inhibited. The expression of OPN (Figure 2D,E) and RUNX2 (Figure 2D,F) in MC3T3-E1 cells also decreased after 7KC administration. This suggested that 7KC could attenuate the osteogenic differentiation of MC3T3-E1 cells. 

### 3.3. 7KC Induced Oxiapoptophagy in MC3T3-E1 Cells

**Figure 3:**
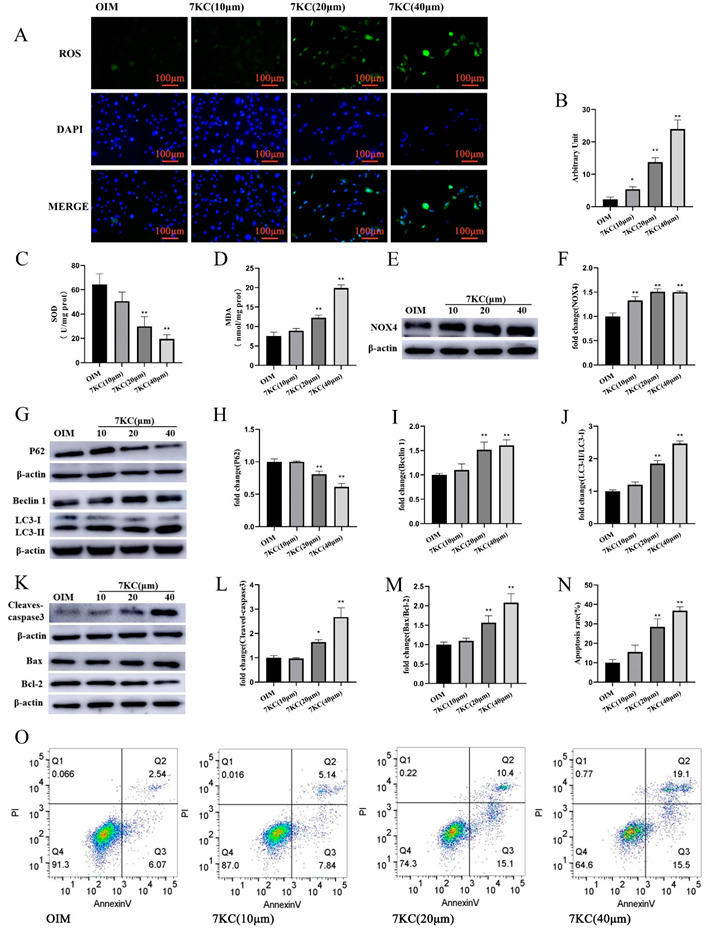
7KC regulated oxiapoptophagy in MC3T3-E1 cells. (**A**) Cells were prepared for staining with DCFH-DA and DAPI, respectively, and analyzed by a fluorescence microscope (×200 magnification). (**B**) Quantitative analysis of ROS production. (**C**,**D**) SOD activity detection, and MDA level measurement were conducted using the kits. (**E**,**F**) The protein expression of NOX4 was analyzed by Western blot. (**G**–**J**) The protein expression of LC3I/II, Beclin1, and P62 was detected. (**K**–**M**) The protein expression of Bax/Bcl-2 and cleaved caspase-3 was measured. (**N**,**O**) Apoptosis analysis was conducted by flow cytometry. Data were analyzed and compared with the OIM group. * *p* < 0.05; ** *p* < 0.01. OIM, osteogenic induction medium; NC, negative control.

7KC is proven to produce oxiapoptophagy [19,21,31]. To explore the biological actions of 7KC on MC3T3-E1 cells, DCFH-DA staining was employed, and the results show that 7KC increased ROS levels in MC3T3-E1 cells in a concentration-dependent manner (Figure 3A,B). The SOD activity and MDA level of MC3T3-E1 cells treated by 7KC significantly decreased (Figure 3C,D). NADPH oxidase 4 (NOX4) plays an important role in the production of ROS, and it has been reported that 7KC can increase NOX4 expression in vascular smooth muscle cells [32,33]. Here, 7KC stimulated NOX4 expression in MC3T3-E1 cells (Figure 3E,F). In addition, 7KC increased the ratio of LC3-II/LC3-I protein expression, enhanced Beclin 1 expression, and significantly decreased P62 protein expression (Figure 3G–J). 7KC treatment also resulted in an increased apoptosis-related protein Bax/Bcl-2 ratio and cleaved caspase-3 protein expression (Figure 3K–M). Flow cytometry showed that 7KC could significantly increase the apoptosis of MC3T3-E1 cells (Figure 3N,O). Collectively, treatment with 7KC in MC3T3-E1 cells might induce oxidative stress, autophagy, and apoptosis, known as oxiapoptophagy. 

### 3.4. NAC Antagonized 7KC-Induced Autophagy, Apoptosis, and Differentiation Inhibition of MC3T3-E1 Cells

**Figure 4:**
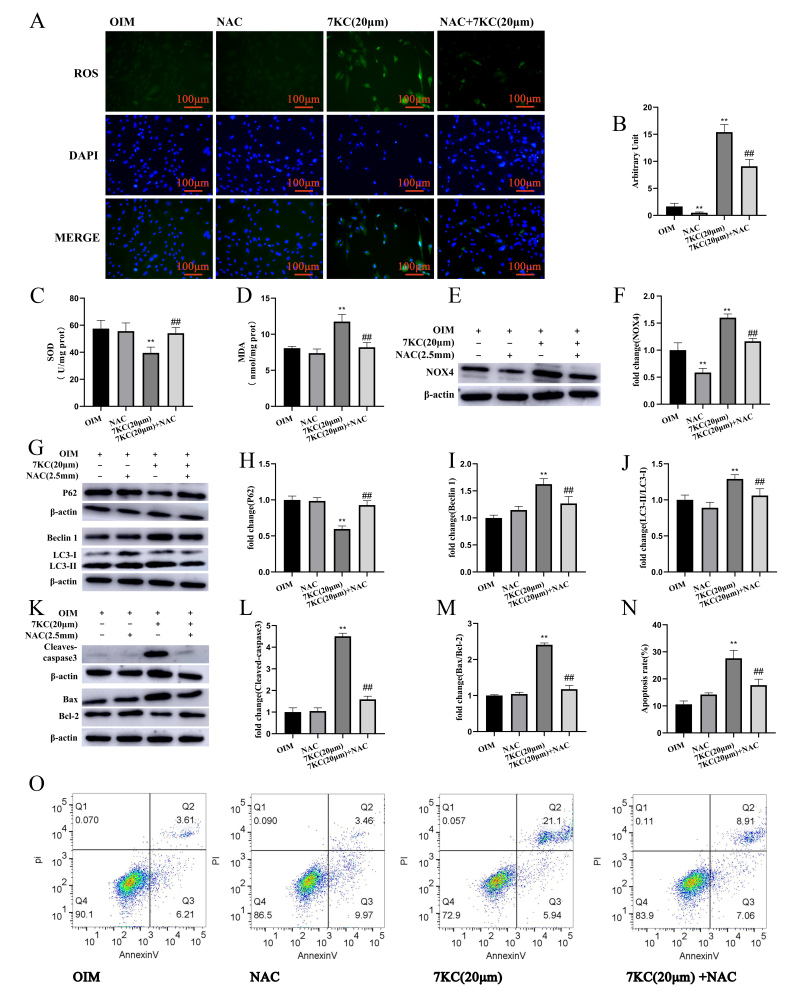

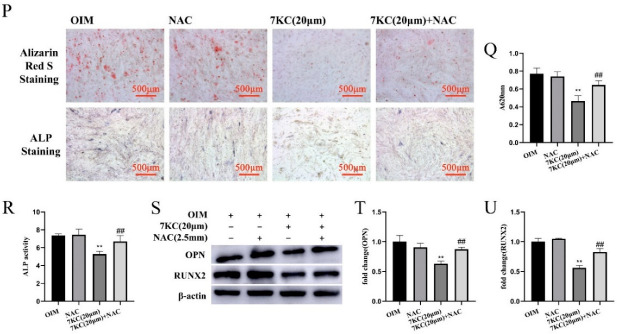
NAC inhibited oxiapoptophagy and differentiation in 7KC-induced MC3T3-E1 cells. (**A**) Cells were prepared for staining with DCFH-DA and DAPI, respectively, and analyzed by a fluorescence microscope (×200 magnification). (**B**) The production of ROS was analyzed by fluorescence intensity. (**C**,**D**) SOD activity detection and MDA level measurement were conducted using the kits. (**E**,**F**) The protein expression of NOX4 was analyzed by Western blot. (**G**–**J**) The protein expression of LC3I/II, Beclin1, and P62 was detected. (**K**–**M**) The protein expression of Bax/Bcl-2 and cleaved caspase-3 was measured. (**N**,**O**) Apoptosis analysis was conducted by flow cytometry. (**P**–**R**) ALP activity detection and the mineral assays were performed (×50 magnification) and analyzed. The protein expression of OPN (**S**,**T**) and RUNX2 (**S**,**U**) was determined by Western blotting. Data were analyzed for comparison with the OIM group. ** *p* < 0.01. Compared with the 7KC (20 μM) group, ## *p* < 0.01. OIM, osteogenic induction medium; NAC, 2.5 mM NAC.

To explore the role of oxidative stress in 7KC-induced oxiapoptophagy and differentiation inhibition, a ROS scavenger (NAC) was added. The results show that NAC preincubation can decrease the ROS levels in 7KC-induced MC3T3-E1 cells, decrease the NOX4 protein expression, increase the SOD activity, and decrease the MDA production (Figure 4A–F). NAC can ameliorate the increased ratio of LC3-II/LC3-I, increase the expression of Beclin 1, and decrease the expression of P62 induced by 7KC (Figure 4G–J). NAC preincubation can also decrease the protein expression of BAX and Cleaved caspase-3 and increase the protein expression of anti-apoptotic protein Bcl-2 (Figure 4K–M). Flow cytometry detection results indicate that NAC preincubation can significantly reduce the apoptosis of 7KC-treated MC3T3-E1 cells (Figure 4N,O). Furthermore, NAC improves the osteogenic differentiation inhibition of MC3T3-E1 cells by 7KC, as shown by the increased activity of alkaline phosphatase and formation of mineralization (Figure 4P-R) as well as the increased protein expression of OPN (Figure 4S,T) and RUNX2 (Figure 4S,U).

## 4. Discussion

Previous studies indicate a correlation of AS with an enhanced fracture risk for bone [5]. However, the biological action of oxysterol, a critical regulator implicated in the pathophysiological process of AS, on bone metabolism has not been fully elucidated. Current results indicate that 7KC lowered the viability, increased the generation of ROS, induced oxiapoptophagy, and attenuated the osteogenic differentiation in pre-osteoblast MC3T3-E1 cells. In addition, pre-incubation with NAC strongly attenuates 7KC-induced pathological changes.

Oxysterols are considered to be a class of factors associated with human physiological and pathological processes. For example, oxysterols were recently demonstrated to function as the ligands that interact with the liver X receptors (LXRα/β), which are involved in the regulation of cholesterol metabolism by mediating the transcription of specific genes, such as ABCA1, ABCG1, and SREBP-1c [34]. Oxysterols may exhibit pro-apoptotic and pro-autophagic activities, depending on the difference in oxysterol types, cell lines, and treatment concentrations [35]. The main types of cell death induced by oxysterols, apoptosis, autophagy, and necrosis, have been studied [10,36]. However, cell necrosis may only be induced by high concentrations of oxysterols in some cell lines [37]. It has been demonstrated that 27-hydroxycholesterol may decrease osteoblast differentiation, increase osteoclastogenesis, and increase bone resorption by interacting with estrogen receptors and LXRs [38]. 25-Hydroxycholesterol is reported to increase apoptosis, promote osteogenic differentiation, and induce vascular calcification in vascular smooth muscle cells (VSMCs) by triggering endoplasmic reticulum stress [39]. 7KC is reported to promote cell apoptosis in mesenchymal stem cells derived from adipose tissue [40]. Similarly, 7KC can induce oxiapoptophagy in BMSCs from patients with acute myeloid leukemia by mediating the Sonic Hedgehog pathway [19]. However, whether 7KC affects osteogenic differentiation by inducing oxiapoptophagy is still unclear.

To investigate the toxicity of 7KC on MC3T3-E1 cells, we measured the effect of 7KC on cell viability. We found that 7KC exhibited cytotoxicity at a concentration as low as 5 μM. At concentrations of 20 μM and 40 μM, 7KC induced apoptosis by 26.3% and 35.6%, respectively. It is reported that 7KC can induce cytotoxicity at a concentration range of 5 to 80 μg/mL in different cell lines in 48 h [41]. In U937 cells, 7KC induces cell apoptosis with maximal proportions (40 ± 5%) at the concentration of 40 μg/mL in 30 h [42]. In VSMCs, 7KC promotes cell apoptosis at a high concentration of 30 μM in 6 days [43]. In human umbilical venous endothelial cells, 7KC at concentrations of 10 μg/mL and 20 μg/mL does not produce effects on cell apoptosis. At a concentration of 40 μg/mL, however, 7KC could significantly induce DNA fragmentation in 20 h [44].

Furthermore, we found that 7KC induced oxiapoptophagy in MC3T3-E1 by up-regulating NOX4 expression and increasing intracellular ROS, thus reducing the survival rate of MC3T3-E1 cells. The presence of the ROS scavenger NAC could compromise these alterations. ROS are produced in cells under various stresses [45]. However, the excessive production of ROS can destroy the balance between oxidant and antioxidant systems and altered ROS generation can lead to the oxidative-stress-induced OP by promoting lipid peroxidation, reducing antioxidant enzyme expression, inducing osteoblast apoptosis, and inhibiting bone formation [46,47,48,49]. It was initially found that 7KC can increase the expression of NOX4, contributing to the overproduction of ROS in human aortic smooth muscle cells [50]. Consistently, 7KC enhances ROS production, promotes the translocation of NOX components from the cytosol to the cellular membrane, inhibits HO-1 expression, and stimulates lysozyme release in human neutrophils [51]. In human red blood cells, 7KC is reported to activate NOX and induce eryptosis by mediating Rac GTPase and PKCζ [52]. NOX4 is an important source of ROS production and is highly expressed in MC3T3-E1 cells [53]. NOX4 has been involved in the glucocorticoid-induced apoptosis of MC3T3-E1 cells [54]. It was found that simvastatin can protect osteoblasts against H2O2-induced oxidative damage by inhibiting NOX4 expression [55]. In addition, iron overload can stimulate ROS generation, enhance NOX4 expression, and induce apoptosis in MC3T3-E1 cells. NAC can scavenge ROS, inhibit NOX4 expression, and ameliorate iron-overload-induced apoptosis [56].

Oxidative stress can inhibit osteoblast differentiation. Although bone and vascular cells share common processes of osteogenesis and mineralization, reduced bone mineralization (osteoporosis) is associated with increased calcification in humans and mice [57,58]. These paradoxical results in skeletal and vascular niches suggest that different internal signaling pathways orchestrated by tissue-specific microenvironments control the production of mineralization in different ways [59]. Mechanically, this discrepancy might be related to the totally different actions of oxidative stress on RUNX2 expression, which is an important regulator of osteogenic differentiation and mineralization in osteocytes and VSMC [57,60]. Growing evidence demonstrates that oxysterols play a critical role in vascular calcification [6]. Studies have shown that 7KC is also highly involved in PI-induced calcification of vascular smooth muscle cells by inducing lysosomal dysfunction and apoptosis [43,61,62]. A study found that 7KC promotes VSMC cell calcification through lysosomal dysfunction-dependent oxidative stress [43]. Additionally, 7KC may play a key pathogenic role in atherosclerotic plaque calcification development [63]. Our study showed that 7KC inhibited the ALP activity and OPN and RUNX2 expression, thereby inhibiting the osteogenic differentiation of MC3T3-E1 cells. However, these effects could be attenuated by NAC.

Bone homeostasis is maintained by the balance between bone formation and bone resorption. Osteoblasts are differentiated from BMSCs and exhibit critical roles in bone formation. Osteogenic differentiation is complex and orchestrated by various signaling pathways, such as Wnt/β-catenin, TGFβ/BMP, MAPK, and Notch signaling pathways [64]. Early osteogenic biomarkers, such as ALP, RUNX2, and SP7, and late biomarkers, such as OPN and OCN, have been used to evaluate the biological effects of potential candidates [65]. RUNX2 is an important transcription factor mastering osteogenic differentiation that regulates the transcriptional expression of these biomarkers [66,67]. ALP, RUNX2, and OCN build a transcriptional network, governing osteogenesis. In the current study, these biomarkers were purposely employed to evaluate the effects of 7KC on osteogenic differentiation. However, their potential mechanisms still need further investigation. Additionally, although our study shows that 7KC can induce MC3T3-E1 oxiapoptophagy and osteogenic differentiation inhibition, these processes are related to the excessive production of ROS. However, the relationship between oxidative stress, apoptosis, autophagy, and osteogenic differentiation was not addressed in detail. More careful studies are required in the future.

## 5. Conclusions

7KC, an oxysterol formed by autoxidation, is involved in the pathophysiological processes of many diseases, such as cardiovascular, neuronal, and retinal diseases. In this study, we observed that 7KC significantly enhanced oxidative stress, induced autophagy, and promoted apoptosis in MC3T3-E1 cells. Meanwhile, 7KC attenuated the expression of osteogenic biomarkers, such as ALP, RUNX2, and OPN, indicating inhibitory activity against osteogenic differentiation. It is important to explore the association of oxysterols, particularly 7KC, with the metabolism of osteoblasts/osteoclasts in bone tissues. Oxysterols can be potent intermediates for the synthesis of steroid hormones or bile acids. Additionally, exogenous or endogenous oxidized cholesterol products are easily accessible. The accumulation of these oxysterols, such as 7KC, may exhibit adverse effects. It is essential to understand the underlying regulatory mechanism of oxysterols as they might become potential targets for the therapeutic management of diseases, including OP.

## Data Availability

The data used to support the findings of this study are included within the article.
